# Understanding Polarizing Community Perspectives on Harm Reduction Strategies: Challenges to Addressing the Opioid Crisis in Appalachian Pennsylvania

**DOI:** 10.13023/jah.0304.07

**Published:** 2021-10-25

**Authors:** Stephanie L. Creasy, Jessica R. Thompson, Christina F. Mair, Jessica G. Burke

**Affiliations:** Department of Behavioral and Community Health Sciences, Center for Social Dynamics and Community Health, University of Pittsburgh Graduate School of Public Health, Pittsburgh PA

**Keywords:** Appalachia, opioid treatment, harm reduction, mixed methods, MAT, pharmacotherapy

## Abstract

**Introduction:**

Rural communities face barriers to opioid treatment and overdose prevention including concerns about stigma and lack of harm reduction services.

**Purpose:**

The aim of this study was to explore community perspectives and understanding of harm reduction approaches to opioid use and overdose in a high-risk Northern Appalachian case community in Pennsylvania.

**Methods:**

A small town approximately 10 miles from Pittsburgh was identified as the community with the greatest predicted probabilities of epidemic outbreak using posteriors from spatial models of hospitalizations for opioid use disorders. We interviewed 20 key stakeholders in the case community in using a semi-structured interview guide and analyzed the qualitative data using an inductive grounded theory approach.

**Results:**

Our findings illustrate how conflicting perspectives about opioid dependence lay the foundation for the polarizing community perspectives on addressing opioid use and overdose and general disagreement regarding the legitimacy of harm reduction approaches versus abstinence-based recovery plans. Community members shared varying perspectives on multiple aspects of the opioid epidemic, including appropriate strategies, treatment, and overdose prevention methods and how community leaders and organizations should respond.

**Implications:**

Opinions, coupled with a general lack of education regarding opioid use and harm reduction options, make it challenging for small communities with limited resources to create comprehensive plans to address the opioid crisis.

## INTRODUCTION

Nonmedical prescription opioid use is concentrated in areas of the U.S. with large rural populations, including Appalachian PA.^1^–^3^ Similarly, the Appalachian region has substantially higher overdose mortality rates compared to the non-Appalachian U.S.^4^ To curtail the morbidity and mortality associated with problematic opioid use (opioid use disorder, or OUD) and overdose, a wide spectrum of OUD care and overdose prevention options exist, ranging from abstinence-based recovery to harm reduction strategies. Rural communities face barriers to opioid treatment from lack of treatment services, stigma and privacy concerns, increased economic deprivation, geographic barriers, and greater availability of opioids compared to urban areas.^2^,^5^ In addition to barriers created by systemic poverty in the region, distinctive characteristics of Appalachia affect the provision of treatment, including lack of access to health professionals or training in evidence-based treatment approaches.^3^–^5^

Medications for opioid use disorder (MOUD), including methadone, buprenorphine, and naltrexone, is evidence-based treatment for people with OUD that can reduce cravings for opioids and withdrawal symptoms, among other benefits.^6^ MOUD is known to be effective at reducing opioid use disorder and overdose.^7^ This harm reduction strategy has been shown to improve patient survival, increase treatment retention, increase patients’ ability to sustain employment, and decrease illicit opiate use and other criminal activity.^5^,^8^,^9^ However, MOUD is underutilized across the country, particularly in rural and non-urban areas and the Appalachian region^4^; for example, Kentucky, Tennessee, and West Virginia are three states that delayed use of MOUD and only recently began covering methadone treatment in their state Medicaid programs.^4^,^10^ The dearth of community providers who deliver MOUD causes barriers for individuals seeking this form of treatment.^11^ Additionally, lack of education about harm reduction strategies, the stigma of “substituting one drug for another,” and misunderstandings of side effects lead to underutilization of MOUD even when available.^12^–^15^

In addition to MOUD, expansion of access to Naloxone was identified as a priority area for addressing the opioid crisis by HHS. Naloxone, an “opioid antagonist” used to quickly reverse the effects of opioid overdose, is one strategy to address opioid overdose prevention among a spectrum of harm reduction strategies, including syringe services programs (SSPs), fentanyl test strips, safe injection sites, housing first programs, and safer use education. Despite evidence of decreased opioid overdose deaths when Naloxone is available and administered, particularly in prehospital settings, the intervention remains underutilized outside metropolitan areas.^12^ Many areas are equipping emergency medical service (EMS) workers, police officers, professional organizations, and family members of individuals at risk for overdose.^12^ However, the effectiveness of efforts to improve Naloxone access, as well as perceptions of this expansion, in rural and non-urban areas is unknown.

Structural barriers and the lack of education regarding the effectiveness of MOUD and harm reduction strategies like Naloxone may contribute to communities not allocating resources to these harm reduction approaches. Indeed, structural barriers to MOUD (e.g., transportation and time) are associated with disfavor for methadone treatment.^16^ Based on our findings, community beliefs around the success of abstinence-based recovery may reduce support for these harm reduction approaches. The view that MOUD and other harm reduction strategies do not render the individual “drug-free” conflicts with the treatment approaches of abstinence-based recovery programs, such as the 12-step program and recovery houses that require sobriety and may be associated with beliefs that MOUD is not appropriate treatment.^16^

While rural areas, particularly those in the Appalachian region, underutilize MOUD and Naloxone in their opioid treatment and overdose prevention efforts,^4^ little information exists on use of these harm reduction approaches in non-urban areas near metropolitan centers (e.g., micropolitan areas or small towns in outlying or fringe counties from urban centers). It is unclear how social factors, such as community beliefs, influence the use and expansion of harm reduction modes of treatment and overdose prevention in communities where physical access may be less of a barrier than in rural areas but community characteristics (e.g., cultural practices or beliefs, socioeconomic factors, and access challenges) may greatly vary from urban settings. Furthermore, little is known about the role of community perspectives on implementation of these treatment options, such as mandated treatment and/or incarceration, or the role of police. Qualitative assessment of community beliefs is vital to informing interventions targeting opioid use in specific geographic locations. Therefore, the aim of this study is to explore community perspectives of MOUD and Naloxone, harm reduction approaches to opioid dependence and overdose, in a high-risk Northern Appalachian case community in Pennsylvania (PA).

## METHODS

### Theoretical Framework

The Social–Ecological Model was used to ground the work contextualizing the opioid crisis in Appalachian PA. The attributional theory of stigma, which posits the cause of a health problem is controllable or reversible by the individual, was used to focus the analysis. This perspective promotes a stigmatizing response to the individual who uses opioids.^17^ This is also evidenced by the language used by some researchers and treatment providers who describe “addiction” as a choice compared to a disease.^17^ Understanding participant responses through these theoretical perspectives connects the multi-layered stigma around those who use opioids to the lack of policies, treatment options, and community support.

### Case Community Selection

Zone Improvement Plan (ZIP) code–level spatial random effects model of hospitalizations for opioid use disorders was used (i.e., opioid abuse and/or dependence) in the state of PA from 2004 to 2014 (16,275 space–time units) to identify communities with the greatest predicted probabilities of epidemic outbreak.^11^,^18^ A discrete target area (i.e., case community) consisting of one Appalachian ZIP code with a population of 100+ was selected using results from those models in combination with the crude opioid use disorder (OUD) hospitalization rate for each ZIP code in 2014 according to ICD-9CM diagnoses from patient-level records of hospitalizations from the Pennsylvania Healthcare Cost Containment Council (PHC4).^19^ The case community, chosen among the identified communities due to its proximity to the study team, is located approximately 10 miles outside of Pittsburgh and has a population size around 4500 people, is 1.6^2^ miles, 94% white, with 15.8% of the population living below the poverty line and a household median income of $31,681.^20^

### Sample Recruitment

Twenty key stakeholders in the case community were interviewed, including clinicians, treatment providers, and other interventionists (seven participants); residents who currently use or have a history of opioid use (eight participants); community leaders (e.g., mayoral staff, police—two participants); and family members of those with a history of opioid use (three participants). Stakeholders were identified through snowball sampling, with a goal of identifying drivers of the opioid epidemic, existing resources, and intervention opportunities within the community by speaking to a diverse range of individuals.

### Data Collection

A research assistant with qualitative data collection training and experience conducted in-depth interviews using a semi-structured field guide previously developed in a pilot study that informed this research by the PI of both studies and a research assistant with qualitative research experience. The field guide was adapted by the PI and the research assistant of this study to reflect changes to the aim of this research. The interviews were approximately 1 hour, and participants were provided $40. The interviews were conducted in-person at an agreed-upon community site and time (e.g., coffee shop) and were audio-recorded. The field guide included semi-structured, open-ended questions on identifying perceptions regarding the range of contextual factors that influence the opioid epidemic in the community and specific attention to perceptions around the scope of the epidemic and how it changed over the past decade; health consequences associated with injection opioid use; drug availability (e.g., where do the drugs come from and how do people who use get them?); and a range of contextual factors, including social and structural factors (e.g., illegal sources), that influence OUD and overdose among residents. The interviews also focused on identifying formal and informal strategies and interventions. In this paper, the focus is on participant perspectives on treatment and harm reduction strategies; additional results on community and social factors are reported elsewhere.^21^ All research was approved by the University of Pittsburgh IRB (PRO16080389).

### Data Analysis

All interview audio recordings were transcribed. Transcripts were read in entirety and imported into the qualitative data management software NVivo 12^22^ for analysis using the inductive grounded theory approach.^23^ Three study team members, including the PI, research assistant, and graduate student researcher, all of whom have extensive qualitative research experience, met weekly to develop the codebook. All segments of text within each interview addressing a priori thematic areas were indexed under a common heading. The team began by using the components of the interview field guide (e.g., changes in opioid epidemic over time) and then expanded with more specific codes identified as a result of the review and analysis process, such as themes covering “different opinions regarding treatment” and “crime and its relationship to use.” The team double-coded 30 percent of the interviews to ensure inter-rater reliability and reviewed and resolved all coding discrepancies. Upon completion of coding, all team members reviewed transcript excerpts from the codebook to identify emergent themes related to our research.

## RESULTS

Conflicting perspectives about opioid dependence lay the foundation for the polarizing community perspectives on opioid treatment and general disagreement regarding abstinence-based recovery versus harm reduction approaches, as well as the role of stigma and police involvement in shaping views and access to harm reduction approaches to treatment.

### Perceptions of Opioid Dependence

The concept of opioid dependence was understood differently among participants, where some discussed individual choices or personal experiences motivating use while others identified structural influences. Some participants, including people with a history of opioid use and treatment providers, communicated that the problems associated with opioid use and dependence (often referred to in communities as “addiction”) were the direct result of an individual’s opioid use rather than a cause or driver of use. However, other participants viewed the issue from a broader perspective shared their beliefs that opioid use is influenced by external community factors and pervasive structural problems. Participants referenced economic change and disadvantage, community isolation, lack of employment opportunities and social spaces, and the high prevalence of mental illness as facilitators for high opioid use within the community:

*Because if you know anything about [this area]… you would think it’s almost like in a war zone. You see all these abandoned houses in the community. You see lots with grass growing six feet high. You just see trash in the community. It’s just a depressing area. And when people live in a depressed area, they have a tendency to become depressed themselves. And depression leads to something to anesthetize themselves, something like a drug or alcohol just to get through a day, you know? (Interview 13, service provider)*

Discussions regarding reasons for use, as well as problems resulting from use, were divergent in nature and typically placed the onus for addressing opioid use either on the person who uses opioids or external factors, but rarely both. Similarly, understanding of opioid use fell into two schools of thought: OUD as an illness or lifestyle. Beliefs about opioid use as a lifestyle were often moral or ethical in nature; beliefs about use as inherently “wrong” meant individuals with OUD and/or those who have experienced an overdose were less deserving of the quality or quantity of treatment offered for other behavioral health conditions. Some participants reported hearing others in the community expressing these views:

*And so, I just think that there’s not enough people that really want it to go away on both sides… And you got people that are now saying, “Hey, forget Narcan. Let him die.” Wow…To let a human being – just to say that is crazy to me, however this person may die, or not die, or be dying, I don’t know. But to say that, “Just let him die,” that speaks volumes about our country right now or our humanity. (Interview 5, community leader)*

### Perceptions of Opioid Treatment Approaches

Most participants were split between two schools of thought regarding treatment: abstinence-based recovery and harm reduction approaches such as MOUD. It was rare for a participant to support the implementation of both treatment modes within this community. Many participants also expressed a lack of knowledge regarding MOUD and other harm reduction strategies. Some participants who were aware of MOUD as a treatment option expressed disapproval from reduced focus on sobriety or “getting clean.”

**Detox**. Participants beliefs on how to address the epidemic in the community differed widely. Some participants stated that availability of any treatment in the community, including detox, is an important missing piece; other participants did not believe detox programs are effective treatment mechanisms for OUD:

*The detox doesn’t seem to be so effective… You feel better about yourself and then you go right back to using because really you haven’t been clean long enough for your mind and everything to heal. It’s just a long process. You used all those years, you’re not just going to get better in 30 days. (Interview 15, resident with a history of use)*

**Medications for opioid use disorder (MOUD)**. Like opinions on detox, participants had strong beliefs about strategies employing harm reduction. Many participants understood harm reduction MOUD approaches, Methadone being the most recognized, as an extension and continuation of opioid use, or “not clean”:

*For me, any type of opiates in your body is still addictive. And so, I’m an old-fashioned recovery type of guy … You still have the same behavior, the nodding. And if you come into a meeting off the medicine when I’m clean, and that’s a trigger for me. (Interview 11, resident with a history of use)*

Understanding MOUD methods as “still addictive” often led participants to describe a tension between harm reduction and abstinence-based recovery. Several participants in the study recounted situations in which individuals attempting to participate in Narcotics Anonymous (NA) meetings or live-in recovery houses were forced away by peers or leaders:

*The recovery community as a whole has a very shitty attitude towards methadone and suboxone. I’ve seen people chased out of the rooms of 12-step recovery meetings and recovery because… wanted to do methadone or suboxone… they say, ‘Oh, you’re still addicted.’ (Interview 12, resident who currently uses)*

**Naloxone**. Like the use of MOUD as a mechanism for addressing the opioid crisis, many participants disagreed about use of Naloxone (Narcan)on individuals who overdose in the community. Some participants agreed with the practice, and some did not:

*I’ve heard both sides of it. It seems that anybody that has had experience with opioids themselves obviously is for the use of Narcan. And… people that are more so conservative… they might say, “Well, if you shoot this Narcan then this junkie is just going to get up and rob my grandmother the next day. (Interview 12, resident who currently uses)*

Further, several participants identified discomfort with using limited community resources on Naloxone as an intervention for opioid overdose, particularly when individuals need intervention multiple times:

*We have police responding to overdoses almost every day. I think that’s taken away from a lot of our elderly that might need that same medical attention or – I still have a problem with all the Narcan that’s being used. If my mother’s having a heart-attack, and the police are going for the third time the same day to save someone who’s overdosing. And that’s medical attention that somebody else is not getting…(Interview 4, resident involved in drug use prevention program)*

**Policing as a Strategy**. Like the use of MOUD as a mechanism for addressing the opioid crisis, the use of police, arrest, and incarceration, as well as mandated treatment as a result of interaction with the criminal justice system, was polarizing for participants. Some participants adamantly believed more police involvement was necessary to curb opioid use in the community. One participant felt individuals arrested for using opioids get off too lightly, which does not “stop” use:

*And most of the people that get caught with heroin… get a pat on their hand. A pat on the hand… And they doing it again. And it’s like they ain’t stopping them. They doing nothing to stop them. (Interview 11, resident with a history of use)*

Other participants, including members of local law enforcement, believed jailing individuals for using opioids would not solve the problem. Likewise, many participants disagreed about police use of Narcan on individuals who overdose in the community. Some participants agreed with the practice, and some did not:

*I’ve heard both sides of it. It seems that anybody that has had experience with opioids themselves obviously is for the use of Narcan. And… people that are more so conservative… they might say, “Well, if you shoot this Narcan then this junkie is just going to get up and rob my grandmother the next day. (Interview 12, resident who currently uses)*

There was no clear consensus on police presence as a tool for arrest, treatment, or both. Some individuals believed if a police officer responded to an overdose and used Naloxone that the individual should be arrested and, perhaps, forced into treatment.

**Multiple or combined approaches**. Participants rarely expressed desire for using both harm reduction and abstinence-based recovery programs. The few participants who did support a multi-layered approach, either individually or as a community practice, felt combining MOUD, NA, and/or recovery homes was helpful:

*I’m clean now. I’m on methadone. It helps. A lot of people were against it, but I think it helps. It’s better than shooting dope and chasing it every day, and I feel like I’m just better on it for now as long as I use it how it’s supposed to. I don’t want to be on it the rest of my life. I have a goal, a therapist. I do treatment. I go to groups. I do the meetings every night. I have a sponsor. I have a home group. I do all that. (Interview 15, resident with a history of use)*

## IMPLICATIONS

Participants in this study had a wide range of perspectives about why OUD and opioid overdose are prevalent in their community, as well as varying opinions about the best treatment and overdose prevention methods for residents with OUD and/or experiencing an overdose. Viewing OUD as an illness versus a lifestyle may cause factions among those interested in working to address the crisis. While some participants identified structural- and community-level factors impacting use, others believed individual opioid use was the cause, rather than a result, of the community’s problems. Residents’ beliefs about opioids, the people who use them, and appropriate treatment tap into moral and ethical perspectives. Most participants were split between two schools of thought regarding treatment: abstinence-based recovery and harm reduction approaches such as MOUD. It was rare for a participant to support the implementation of both treatment modes within this community. Many participants also expressed a lack of knowledge regarding MOUD and other harm reduction strategies. Some participants who were aware of MOUD as a treatment option expressed disapproval from reduced focus on sobriety or “getting clean.” These views align with notions found in previous literature on perceived negative side effects and avoidance of using MOUD, which may be influenced by policy and social norms.^24^–^26^

Diverse community perspectives, stigma of harm reduction strategies, and lack of knowledge of evidence-based interventions may make it difficult for small communities with few resources to implement a comprehensive plan to address OUD and overdose in their communities. In addition, community leadership may face challenges in acquiring funding and support for their constituents’ needs, particularly when there is profound disagreement and lack of clear guidance regarding effective community-level interventions. These systemic and community-level challenges have been previously documented as particular barriers in rural and non-urban communities, where resource constraints are often greater.^25^,^27^,^28^ Future public health education should provide knowledge of evidence-based approaches, as well as advocate for less dichotomy and either/or ideologies in the OUD treatment and overdose prevention spheres, particularly in economically disadvantaged areas such as the case community in this study. Community plans for opioid treatment could incorporate multi-level interventions that provide education and access to both harm reduction strategies and abstinence-based support, following the example of the multimodal treatment approaches introduced and advocated for in the mental health literature.^29^–^31^

Similarly, the role of police and the criminal legal system is another challenging area of disagreement for communities facing an opioid crisis. These results reflect previously identified barriers to Naloxone acceptance (e.g., cost, legality, and lack of knowledge of distribution) and suggest the need to uncover ways to implement policies and increase harm reduction strategies while reducing stigma and mistrust.^32^,^33^ One promising harm reduction intervention is the law enforcement assisted diversion (LEAD) program that diverts individuals from the criminal legal system to case management programs for a range of support services; LEAD is associated with lower odds of arrest and felony charges and is gaining support across the country since its launch in 2011.^34^ However, disagreement within the community regarding the role of police and the effectiveness of criminalization, mandated treatment, and the use of Naloxone makes it difficult for effective community planning and programming, calling for additional analyses around these outcomes.

These qualitative findings are limited to non-urban Appalachian PA. The 20 stakeholder participants may not represent the full breadth of community perspectives and may contain bias as a result of the snowball sampling frame. However, these results suggest division in community perspectives may be a factor in other geographic areas with limited knowledge, resources, or access to care. Future research, even in predominantly white communities like this case community, should explore the relationships between race, socioeconomic status, and the different community perspectives of opioid crises and treatment.

Our results highlight the importance of expanding treatment options in smaller communities. Pilot-testing MOUD interventions in this case community, for example, may be an effective next step in understanding how to incorporate evidence-based harm reduction approaches in small communities affected by the opioid crisis.

**SUMMARY BOX**

**What is already known about this topic?** The Appalachian region has substantially higher overdose mortality rates compared to the non-Appalachian United States. Rural communities face barriers to opioid treatment including concerns about stigma and lack of treatment services, and challenges related to polarizing views of OUD and overdose have been prevalent in treatment and recovery spheres for decades, though less frequently discussed in the literature.

**What is added by this report?** Our findings illustrate how conflicting perspectives about opioid use, OUD, and opioid overdose lay the foundation for the polarizing community perspectives on opioid treatment and overdose prevention and general disagreement regarding the legitimacy of harm reduction approaches versus abstinence-based recovery plans.

**What are the implications for future research?** Future public health education should provide knowledge of evidence-based approaches, as well as advocate for less dichotomy in the treatment sphere, particularly in economically disadvantaged areas such as the case community in this study. Community plans for OUD care and overdose prevention could incorporate multi-level interventions that provide education and access to harm reduction strategies within the full spectrum of OUD care.
